# A 3-Arm randomised controlled trial of Communicating Healthy Beginnings Advice by Telephone (CHAT) to mothers with infants to prevent childhood obesity

**DOI:** 10.1186/s12889-016-4005-x

**Published:** 2017-01-14

**Authors:** Li Ming Wen, Chris Rissel, Louise A. Baur, Alison J. Hayes, Huilan Xu, Anna Whelan, Myna Hua, Miranda Shaw, Philayrath Phongsavan

**Affiliations:** 1 Health Promotion Unit, Sydney Local Health District, Level 9, King George V Building, Missenden Road, Camperdown, NSW 2050 Australia; 2 Sydney School of Public Health, Sydney Medical School, University of Sydney, Camperdown, Australia; 3 Charles Perkins Centre, University of Sydney, Camperdown, Australia; 4 Office of Preventive Health, Ministry of Health, Camperdown, NSW Australia; 5 Discipline of Child & Adolescent Health, University of Sydney, Camperdown, Australia; 6 Health Promotion Unit, South Eastern Sydney Local Health District, Camperdown, NSW Australia; 7 Community Health Services, Sydney Local Health District, Camperdown, NSW Australia

**Keywords:** Randomised controlled trial, Childhood obesity, Health promotion, Intervention, Telephone consultation, Text messaging, Infant feeding practice, Breastfeeding, BMI

## Abstract

**Background:**

With an increasing prevalence of obesity in young children globally, there is an urgent need for the development of effective early interventions. A previous Healthy Beginnings Trial using a nurse-led home visiting program has demonstrated that providing mothers with evidence-based advice can improve maternal practice regarding obesity prevention, and can reduce Body Mass Index (BMI) in the first few years of life. However, the costs for scale-up of home visiting limit its population reach. This trial aims to determine the efficacy of Communicating Healthy Beginnings Advice by Telephone (CHAT) to mothers with infants in improving infant feeding practices and preventing the early onset of childhood overweight and obesity.

**Methods/Design:**

We propose a 3-arm randomised controlled trial (RCT) with a consecutive sample of 1056 mothers with their newborn children in New South Wales (NSW) Australia. Pregnant women who are between weeks 28 and 34 of their pregnancy will be invited to participate in the CHAT trial. Informed consent will be obtained, and after baseline data collection, participants will be randomly allocated to the telephone intervention, text messaging intervention, or the control group. The intervention comprises telephone consultations or text messages, together with 6 intervention packages being mailed at specific times from the third trimester of pregnancy until 12 months post birth. The main trial outcome measures include a) duration of breastfeeding, b) timing of introduction of solids, c) nutrition behaviours, physical activity and television viewing, and d) weight and BMI z-score at 12 and 24 months, e) cost-effectiveness, as well as f) feasibility and acceptability of the interventions.

**Discussion:**

The results will ascertain whether early intervention using telephone consultation or text messaging together with staged mailed intervention resources can be feasible and effective in improving infant feeding practices, physical activity and reducing children’s BMI in the early years of life. If proven to be feasible, effective as well as cost-effective, the trial results will inform a series of recommendations for policy and practice related to promoting healthy infant feeding and physical activity in young children in the first years of life.

**Trial registration:**

The CHAT Trial is registered with the Australian Clinical Trial Registry (ACTRN12616001470482p). It was registered on October 21, 2016.

## Background

The importance of early intervention in the first few years of life to prevent the development of overweight and obesity is evident, with a global prevalence of overweight being 6.7% of children under the age of five in 2010 [1]. In Australia, approximately 20% of children at 2–3 years of age are overweight or obese [2]. It has now become evident that excess weight and fast weight gain in young children are associated with overweight later in life [3–6]. Obesity in pre-school aged children has a high risk of progressing to obesity in later childhood [5] and potentially into adulthood [6, 7]. The adverse health consequences of childhood obesity are well documented internationally [8, 9], and in Australia obesity in very young children is associated with increased healthcare utilization [10]. There was a call for greater efforts being made to prevent childhood obesity in the early years, and even before birth [11]. Indeed, a 2013 Lancet editorial argued that prevention, through educating mothers about both nutritional and environmental exposures of in-utero and during early life is an important strategy in the prevention of non-communicable diseases [12].

Of the many factors contributing to early onset of childhood obesity, research has found that infant feeding practices, including breastfeeding [13, 14] and the timing of the introduction of solids [15, 16] as well as children’s eating habits [17] and television (TV) viewing time [18, 19] are among the most modifiable. Infant feeding practices can affect both child eating behaviours and adult eating habits later in life [20]. It was concluded by a systematic review that obesity-related behaviours can be influenced positively in a range of settings [21].

Over recent years, there has been an increasing number of research studies on developing and implementing early obesity interventions [22, 23]. In 2012, we completed the Healthy Beginnings Trial (HBT) [22, 24, 25], which was the first randomised controlled trial (RCT) to test the effectiveness of an early childhood obesity intervention during the first 2 years of life. Briefly, HBT is a staged, home-based early intervention, designed to improve family and behavioural risk factors for childhood obesity. The intervention involved home visits by an early childhood nurse, with an initial visit at late pregnancy, and then seven visits at 1, 3, 5, 9, 12, 18 and 24 months after birth [24]. This early intervention was effective in reducing children’s body mass index and reducing TV viewing at age 2 years [22], as well as improving infant feeding practices including breastfeeding [25]. However, the economic evaluation of the HBT showed that the early obesity prevention through home visits was only moderately cost-effective, with the cost of home visiting potentially limiting the level of population reach [26]. Hence, other new, low cost and high uptake approaches need to be explored.

Over the past 10 years, evidence has emerged rapidly regarding the use of Short Message Service (SMS) and telephone consultation in health promotion interventions, health service delivery and promotion of health behaviour change [27–29]. Telephone consultation often employs a motivational interviewing approach and aims to set goals, build skills and knowledge, and address barriers as well as manage anxiety and stress which may act as barriers to health behaviour change. The SMS is used to reinforce knowledge, prompt use of behaviour change strategies (e.g., goal setting, problem solving, overcoming barriers) and allow users to seek support for management of problems in real time. Telephone-delivered interventions have shown a moderate effect for promoting healthy lifestyles [27], while SMS-delivered interventions have positive short-term behavioural outcomes [29] with strong evidence supporting the value of integrating text-messaging interventions into public health practice [28]. To date, few published studies have examined the effectiveness and cost-effectiveness of telephone- or SMS- delivered intervention in promoting healthy infant feeding practices and obesity-protective behaviours in the early years of life. The evidence to date shows that telephone- or SMS- delivered interventions hold promise as an alternative delivery model for the Healthy Beginnings^©^ early intervention program (http://www.healthybeginnings.net.au/) to mothers with newborns, at lower cost and with greater population reach.

### Aims

The CHAT trial aims to evaluate the efficacy of using SMS or telephone support, plus mailed written intervention materials developed through the HBT, to deliver a staged intervention based upon the principles of the successful HBT intervention. The proposed study design is an RCT with three arms (including mailed HBT intervention materials plus telephone support; mailed HBT intervention plus SMS; and usual care) starting in the 3rd trimester of pregnancy and continuing until the child is 12 months. We will compare each intervention with usual care, and will evaluate the cost-effectiveness of each intervention compared with usual care.

We hypothesise that, compared with usual care, the intervention using either SMS or telephone support will lead to:

(*primary outcomes*)

- an increased breastfeeding rate and duration and appropriate timing of introduction of the solids at 6 months;
- an increased breastfeeding rate and duration at 12 months;
- a reduction in child BMI z-score at 12 and 24 months; and

(*secondary outcomes*)

- an increased rate of practising “tummy time” at 6 months;
- an increased rate of using cup and drinking water at 12 months;
- a reduction in child TV viewing time at 12 and 24 months;
- an increased dietary quality (i.e. an increased intake of fruits/vegetables) of the child at 24 months;
- a reduction in child BMI z-score at 12 months;
- demonstrated cost-effectiveness; and
- demonstrated feasibility and acceptability of the interventions.

More specifically, the proposed study aims to address the following **research questions**:

1. Will an SMS or telephone support early intervention with mailed health resources delivered in the antenatal period and over the first twelve months of life significantly reduce children’s BMI at ages 12 and 24 months, when compared to usual care?
2. Will an SMS or telephone support early intervention delivered with mailed health resources in the antenatal period and over the first twelve months of life significantly improve infant feeding practices at age 12 months, and significantly decrease the prevalence of obesity-related behaviours among children aged 12 and 24 months, compared to usual care?
3. From a health funder perspective what are the incremental costs and incremental benefits of the SMS or telephone support early interventions with mailed health resources to reduce childhood obesity compared to usual care, and is one intervention more cost-effective than the other?
4. Will the intervention be feasible and acceptable and to what extent the interventions are likely to have to be implemented in a real world context?

## Methods

### Design

**Fig. 1:**
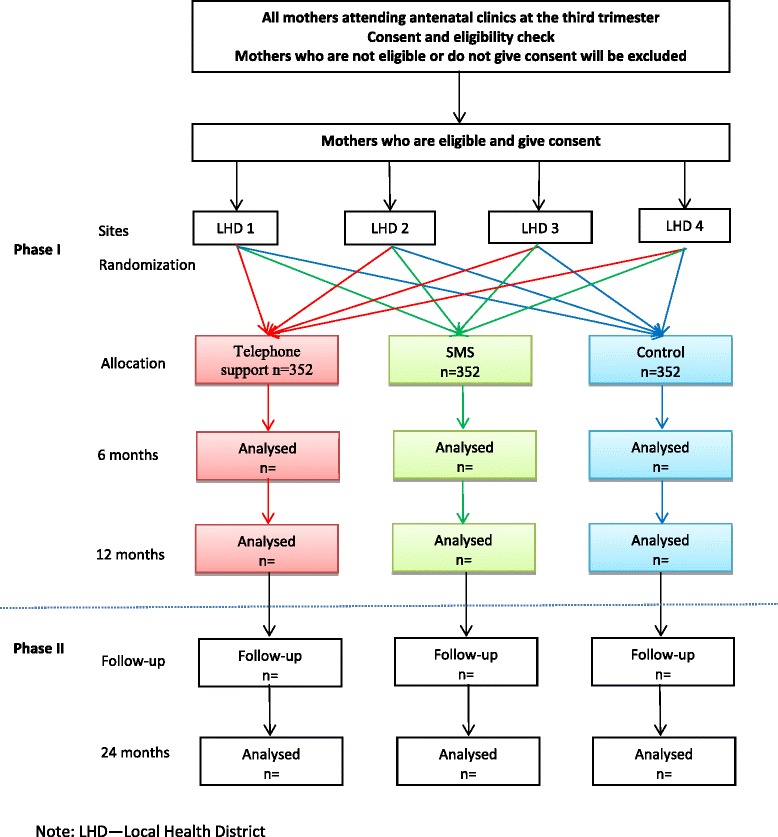
Study design

A 3-arm parallel randomised controlled trial (2 intervention arms and 1 control arm). We plan for two phases of the trial including Phase 1, an intervention phase with outcomes measured at 12 months of age, and Phase 2, a follow-up phase (no further intervention) with outcomes measured at 24 months of age (see Fig. 1).

### Setting

The study will be conducted in the regions covered by the Sydney, South Eastern Sydney, South Western Sydney and Southern NSW Local Health Districts in NSW, Australia.

### Participants and recruitment

All women at 28–34 weeks of pregnancy who attend antenatal clinics of the main hospitals in one of four local health districts will be approached by research assistants (RAs) with a letter of invitation and information about the study. Written informed consent will be obtained from all study participants. Once eligibility is established and consent obtained, women will be asked to fill in a registration form with their contact information to allow the RA to make further arrangements for the baseline data collection via Computer Assisted Telephone Interviewing (**CATI**) and random allocation to an arm of the study.

### Inclusion criteria

Women will be eligible to participate if they are aged 16 years and over, are at 28–34 weeks of pregnancy, are able to communicate in English (or can communicate with written information in Chinese or Arabic), have a mobile phone and live in the recruitment areas.

### Exclusion criteria

At the initial stage, women will be excluded from the study if they have a severe medical condition based on advice given by their doctors. Women who cannot give informed consent, those expecting multiple births (e.g. twins) and those with babies with known major fetal anomalies will also be excluded.

### Sample size

A consecutive sample of 1056 mothers (352 in each arm) will be required for the study. The sample size calculation is based on the primary outcome, mean BMI z-score, with 80% power and two-sided 5% significance level. To detect a difference in mean BMI z-score of 0.29 units between each intervention group and the control at age 2 years, a sample size of 264 per arm is needed, using an estimated SD of 1.19, based on the HBT data. Allowing for a 25% drop-out (based on HBT), a sample size of 352 per arm will be needed, which will also give enough power to detect differences in secondary outcomes (i.e. a difference of 0.3 in mean number of daily serves of fruit or vegetables).

### Registration and randomisation

Information collected from the registration form will be entered into a password-protected database. A letter will be sent by the RA to inform the obstetrician/midwife that the mother is enrolled in the research trial. An appointment for the baseline data collection using CATI will be made with all participating mothers by letter, followed by a telephone call. The baseline data collection will take place within a week of their consent and registration.

Random allocation to one of three groups will be determined by a computer generated random number using random permuted blocks, stratified by the local health district. After baseline data collection, the RA will inform participating mothers of their group allocation by mail or by a phone call.

### Proposed interventions

Based on the principles of the successful Healthy Beginnings^©^ early intervention program, which is a staged early intervention corresponding to the milestones of infant development, we propose an SMS or telephone support together with mailed intervention resources to be delivered in the antenatal period and over the first 12 months of life. Six packages containing the intervention resources (that will be adapted and revised based on the Healthy Beginnings^©^ program) will be posted according to the developmental stage of the main issues at set times (see Table 1). Following each posted intervention package, an SMS or telephone support intervention will be conducted to reinforce the health promotion messages (such as “Breast is best”, “Tummy time is fun”, “No solids for me until 6 months”, “Only water in my cup” and “I eat a variety of fruit and vegetables every day”), and address mothers’ concerns and needs about the healthy beginnings of life and childhood obesity. The development of SMS messages and telephone support guidance will emphasise skills development, problem solving, and identify strategies to overcome barriers. The intervention will also provide psychosocial support to alleviate mothers’ concerns, stress and anxiety. We will employ two Child and Family Health nurses (also referred as intervention/research nurses in this paper) to deliver the telephone support or SMS intervention.

**Table 1:** Key messages and main contents of the staged early interventions

Time	Main issues/key messages	Main contents
3rd trimester	Breastfeeding and/or formula “Breast is best”	Breastfeeding guidelines; health benefits of breastfeeding and strategies to overcome barriers associated with breastfeeding
1 month	Breastfeeding and/or formula “Breast is best” “Tummy time is fun” “No solids for me until 6 months”	Rapid response to women with problems initiating breastfeeding after childbirth, especially women who delivered by caesarean section; advice on establishment of breastfeeding pattern; management of problems; “tummy time” for babies
3 months	Breastfeeding and/or formula Timing of Introduction of solids “Tummy time is fun” “No solids for me until 6 months”	Advice on establishment of breastfeeding patterns; management of problems; “tummy time” for babies; introduction of solids after 6 months; encourage mothers going back to work to continue breastfeeding
5 months	Timing of Introduction of solids Tummy time “No solids for me until 6 months”	Reinforce breastfeeding pattern; management of problems; “tummy time” for babies; introduction of solids from 6 months; encourage mothers going back to work to continue breastfeeding
9 months	Food serving size, cup feeding/Introduction of cow’s milk “Only water in my cup”	Encourage sustained breastfeeding; advice on balanced infant feeding, quantity and variety of fruits and vegetables; encourage active play and motor skills development; avoiding TV viewing, introduce cup feeding
12 months	Healthy food choices (no food rewards)/Active Play/TV time “I eat a variety of fruit and vegetables every day”, “Healthy eating for whole family”	Encourage sustained breastfeeding; advice on balanced infant feeding, quantity and variety of fruits and vegetables; encourage active play and motor skills development; avoiding TV viewing

### Intervention arm 1 (SMS)

We will purchase a 2-way SMS service that allows for some interactivity between mothers and intervention nurses. Following a mailed stage-specific intervention resource, mothers in this intervention group will be sent text messages to their mobile phone automatically at a predetermined time (10 am–1 pm) and twice a week for 4 weeks. The frequency and time of SMS are based on our pilot study. The messages will provide information/advice, motivation/reinforcing and prompting (messages), psychosocial support for healthy infant and child feeding and lifestyle as outlined in Table 1. A bank of standardised but personalised (i.e. participant’s name) text messages will be developed and pilot tested according to the key infant milestones/stages as outlined. The typical length of the message will be <160 characters. The 2-way SMS service will also receive and save text messages from participating mothers in an excel spreadsheet which will be reviewed daily except for weekends and public holidays. Common questions will be answered by return SMS within 24 h (except weekends) by a research nurse.

### Intervention arm 2 (telephone support)

Mothers in this intervention group will also receive 6 staged intervention packages by mail followed by a phone call from the research nurse within 1–2 weeks. At each telephone contact, the nurse will spend approximately 30 min with the mother to go through the mailed intervention resources and discuss the issues or concerns raised by the mother. A telephone support guidance and manual will be developed based on existing materials, containing a checklist to ensure that all information is covered. Telephone calls will follow a tailored principle, and a framework that reinforces information in the resource packages, helps set goals, works with mothers to develop action plans to achieve goals, deals with barriers and problem solving. Over the study intervention period, 6 telephone support sessions will be conducted, which takes into account practicalities and sustainability of the telephone intervention if it is found to be effective. Telephone support sessions will be delivered by a research nurse trained in telephone support strategies.

### Control arm

Mothers in the control group will receive usual care provided by the usual childhood nursing service from Community Health Service nurses in the local districts. To maximise the retention rate, we will post home safety promotion materials and a newsletter on “Kids’ Safety” to the control group 4 times over the study period.

### Retention strategies

We will use retention strategies already shown to be effective including a) a thank-you card for each participating family, b) New Year greeting cards, c) promoting the study’s identity through branding of all study materials with the study logo, and d) obtaining contact numbers of participants’ relatives. In addition, a specially prepared newsletter on “Kids’ Safety” will be sent 4 times a year to the control group.

### Data collection

The methods and measures of data collection are listed in Table 2. The CATI survey will be used for data collection at baseline, 6 and 12 months. Face-to-face interviews will be conducted for data collection at 24 months. The questionnaires will be mainly based on the previous HBT study [22, 24, 25].

**Table 2:** The methods and measures of data collection at baseline and 6, 12, and 24 months of age

Phase	Phase 1 Study			Phase 2 Study
Time	Baseline	6 months	12 months	24 months
Methods	CATI	CATI	CATI	Face-to-face interview
Main Domains/Measures				
Family demographics	X			
Mother’s knowledge and intention regarding breastfeeding	X			
Mother’s dietary behaviours	X		X	X
Mother’s physical activity and screen time	X^a^		X	X
Mother’s height and weight	X^a^		X	X
Breastfeeding		X	X	X
Introduction of solids		X		
Tummy time^b^		X		
Infant feeding practices		X	X	
Parent-child interaction		X	X	
Child’s eating habits			X	X
Child’s physical activity and screen time			X	X
Child’s sleep patterns			X	X
Child’s length and weight		X	X	X

^a^ before pregnancy

^b^ a colloquial term used to encourage parents to ensure that their babies spend time in the prone position

*CATI* computer assisted telephone interviewing

### Main measures and outcomes

#### At baseline

We will collect socio-demographic data including mother’s age, employment status, education level, marital status, language spoken at home, and country of birth, using the standard NSW Health Survey questions [30]. We will also collect mother’s pre-pregnancy height and weight, gestational weight gain and whether gestational diabetes was diagnosed.

#### At 6 months

We will ask mothers about their infant feeding practices including breastfeeding duration and timing of the introduction of solids. For example, mothers will be asked “Is ‘child’ currently being breastfed?” If they answered “no’, they are further asked “Including time of weaning, what is the time ‘child’ is breastfed?” Mothers will be also asked “At what age is ‘child’ first given solid foods regularly?” We will also collect information on “tummy time” (a colloquial term used to encourage parents to ensure that their babies spend time in the prone position) and parent-child interaction.

#### At 12 months

We will collect breastfeeding information as the primary outcome. Other secondary outcomes will include cup usage, bottle at bedtime, and food for reward as well as play time and activities. We will also collect length and weight of the child recorded in the Personal Health Record (also known as the ‘Blue Book’). In addition, we will send a brief online survey monthly via email to all mothers for the first 12 months, so mothers can record their child’s growth milestones and important events of relevance (i.e., breastfeeding, timing of introduction of solids, “tummy time” and cup usage) and weight gain.

#### Outcomes at 24 months

The primary outcome is the child’s BMI z-score. Secondary outcomes include child’s screen time (including TV) and fruit and vegetable consumption as well as other eating habits (e.g. intake of “extra foods”, and having a meal in front of the TV), active play time and several maternal outcomes: dietary behaviours, TV viewing time, physical activity and knowledge and confidence about obesity prevention. Mother’s height and weight will also be measured. Measurements will be taken during a face-to-face interview by a RA.

### Length

Two measurements will be taken supine (using SECA 210 Infant Measuring Mat, Hamburg, Germany) on a level floor by a research assistant and recorded to the nearest 0.1 cm; a third measure will be taken if the first two measurements differ by 0.5 cm or more and the mean will be recorded.

### Weight

Measurements will be taken using digital scales (Tanita model 1583 Baby Scale, Tokyo, Japan) by a research assistant, from children wearing light clothes and no shoes. The measures will be recorded to the nearest 0.1 kg.

### Eating habits

Mothers will report their child’s eating habits using a short food frequency questionnaire (FFQ) that was specifically designed to assess children’s eating habits, and the validity and reliability of which were tested prior to this study [31]. The FFQ includes serves of fruit and vegetables; frequency of eating snack foods (biscuits, cakes, donuts, muesli bars, potato crisps), and cups of soft drinks/cordials, juice, and water, and frequency of eating in front of the TV and having food as reward.

### Screen time and outdoor play time

Mothers will report the total time their child spends on screen activities (including watching TV) or outdoor play time per day in a usual week using a set of validated questions [32].

### Mothers’ nutrition and physical activity

Mothers’ own dietary behaviours and physical activity will be assessed using questions sourced from the NSW Health Survey Program [30] which we have used previously in HBT [22].

### Data analysis

All outcomes will be compared between each of the intervention groups and the control group. All analyses will be conducted by ‘intention to treat’, which will include all the participants who are randomised, according to their group assignment, regardless of their later status, including noncompliance, protocol deviations, withdrawal, and anything that happens after randomisation. For continuous variables, such as BMI or BMI z-score, means will be compared using *t*-tests. For categorical variables such as percentages of children using a cup, chi-squared tests will be used. Survival analysis will be used for breastfeeding duration: Kaplan-Meier curves will be used to estimate median breastfeeding duration and log-rank tests will be used to compare median breastfeeding duration between groups; Cox proportional hazards regression will be used to compare the hazard ratio for stopping breastfeeding between groups.

Mean BMI-, weight- and length-for-age z-scores at 12 and 24 months will be calculated using WHO Anthro [33]. For comparisons between the groups we will take two steps as we used in the original HBT; firstly, unadjusted and complete-case analysis will be conducted and secondly, adjustment for important covariates or potential confounding factors will be conducted when appropriate. Multiple imputation by chained equations will be used to impute missing values. The effect of loss to follow-up will be assessed by comparing complete-case and multiple imputation analyses.

### Assessor blinding

Outcome data at 6 and 12 months will be collected by a market survey company using CATI, and outcome data at 24 months will be collected by RAs not involved in intervention implementation. The data collectors and data entry staff will be blinded to treatment allocation.

### Cost-effectiveness analysis (CEA) of the intervention

We will carry out cost-effectiveness analysis (CEA) alongside the RCT, in which outcomes will be measured in natural units, such as BMI-z-score. This approach is similar to that used in the original HBT [26].

### Analysis of costs

We will collect data prospectively on the costs to deliver each intervention program during the first 2 years of life (including the 2-way SMS service, or telephone calls, staff time, training, mail-outs, and any other intervention resources). We will include the cost of all resources needed to reproduce each intervention, but will exclude the research and development costs of the study. Both fixed costs and variable costs will be identified, thus allowing estimation of total budget required to deliver a scaled up program. The reference year for the analysis will be 2016; all costs in dollars will be indexed to that year using the Health Price Index. All costs and effects will be appropriately discounted. Market prices will be used as appropriate for the cost of all intervention materials.

### Outcomes for CEA

The key outcome measures including breast feeding duration and BMI will be used to assess the cost-effectiveness of each intervention compared with usual care. Incremental cost-effectiveness ratios will be calculated in terms of the incremental cost: a) per additional infant breastfed to 6 months and 12 months; and b) per unit reduction in BMI z-score at 12 and 24 months. Using the mean costs and the mean health outcomes in each arm of the trial, the incremental cost per unit outcome of each intervention compared to usual care will be calculated and results will be plotted on a cost-effectiveness plane. In order to reflect the variation in mean costs and outcomes, bootstrapping will be used to estimate joint uncertainty in these variables, and to calculate the confidence intervals around the incremental cost effectiveness ratio. One-way sensitivity analysis will be conducted around key variables, and a cost-effectiveness acceptability curve will determine the probability of being cost-effective at different willingness to pay thresholds.

### Pilot and process evaluation

Prior to the trial commencement, the intervention materials including mailed intervention packages, telephone support guidance and SMS messages will be developed and pilot tested with intervention nurses and mothers in the community. The intervention nurses and RAs will be trained according to the protocol. At the recruitment stage of study participants RAs will record their recruitment process using field notes. The intervention nurses will document all aspects of their contact (SMS or telephone support) with the families, and provide regular reports to the investigators on questions and issues arising. Information on the number and duration of telephone calls and SMS messages received by participants, data on recruitment, study retention and intervention acceptability will be recorded. We will routinely conduct quality control audits on records and, each quarter, we will conduct in-depth interviews with 5% of the sample to ensure families are satisfied with the processes, and to identify any other issues that may emerge.

### Evaluation of the feasibility and acceptability

We will conduct in-depth interviews or focus groups with some participants to assess program satisfaction, and to identify any issues that may emerge as well as to gather their feedback on the intervention acceptance. In addition, qualitative interviews will be conducted with stakeholders and representatives from the main organisations involved in the trial to gain a better understanding of their experience of the project implementation, acceptability, and barriers and enablers to effective delivery and suggestions for improving the program to be delivered in the broader community. All interviews will be audio-recorded and transcribed verbatim. Framework Analysis [34] will be used to organise and analyse the data thematically.

### Timelines

The study will begin in February 2017, and end in December 2019 for Phase 1 study and December 2020 for Phase 2 study.

## Discussion

A focus on prevention of childhood obesity in the early years is one of the key recommendations from the 2016 Final Report of the WHO Ending Childhood Obesity Commission [35], and has also been identified as one of the 12 Premier’s Priorities in NSW Australia, which aims to reduce overweight and obesity among children by 5% over the next 10 years [36]. However, current evidence of effective early interventions is scarce. Early obesity prevention strategies that are proven to be effective, sustainable and having a broad population reach with low cost have yet to be developed.

In this proposed study we aim to translate the successful evidence from the HBT [22, 25] and evidence from effective telephone- or SMS-delivered health promotion interventions into scaled-up obesity prevention practice. We aim to demonstrate that communicating the Healthy Beginnings advice to mothers with newborns by telephone or SMS can be feasible, effective and cost-effective, which can lead to a low-cost, broad-reach and sustainable program for promoting healthy infant feeding and preventing early onset of childhood obesity. This CHAT trial can inform policy makers of efficient and effective ways of using resources for achieving population reach and equality through its mass reach capability.

The project will determine whether: 1) a staged early intervention starting from the antenatal period can improve infant feeding practices, child diet quality, screen time and physical activity, and therefore child BMI in the first years of life; and 2) a mobile phone SMS intervention or telephone support is a well-accepted, cost-effective and viable strategy for disseminating knowledge, and promoting healthy behaviour, to mothers living in various geographical locations. If the interventions prove to be successful, we anticipate immediate public health benefits and policy/practice recommendations for preventing the early onset of childhood obesity, which will provide an urgently needed model for population-based, low-cost childhood obesity prevention programs.
